# Feasibility and results of a randomised pilot-study of pre-discharge occupational therapy home visits

**DOI:** 10.1186/1472-6963-7-42

**Published:** 2007-03-14

**Authors:** Natasha Anne Lannin, Lindy Clemson, Annie McCluskey, Chung-Wei Christine Lin, Ian D Cameron, Sarah Barras

**Affiliations:** 1 Rehabilitation Studies Unit, The University of Sydney, PO Box 6, Ryde, New South Wales 2112, Australia; 2 Faculty of Health Sciences, The University of Sydney, PO Box 170, Lidcombe, New South Wales 1825, Australia; 3 School of Physiotherapy, The University of Sydney, PO Box 170, Lidcombe, New South Wales 1825, Australia; 4 Donvale Rehabilitation Hospital, Ramsay Healthcare, PO Box 1058, Surrey Hills North, Victoria, 3127 Australia

## Abstract

**Background:**

Pre-discharge home visits aim to maximise independence in the community. These visits involve assessment of a person in their own home prior to discharge from hospital, typically by an occupational therapist. The therapist may provide equipment, adapt the home environment and/or provide education. The aims of this study were to investigate the feasibility of a randomised controlled trial in a clinical setting and the effect of pre-discharge home visits on functional performance in older people undergoing rehabilitation.

**Methods:**

Ten patients participating in an inpatient rehabilitation program were randomly assigned to receive either a pre-discharge home visit (intervention), or standard practice in-hospital assessment and education (control), both conducted by an occupational therapist. The pre-discharge home visit involved assessment of the older person's function and environment, and education, and took an average of 1.5 hours. The hospital-based interview took an average of 40 minutes. Outcome data were collected by a blinded assessor at 0, 2, 4, 8 and 12 weeks. Outcomes included performance of activities of daily living, reintegration to community living, quality of life, readmission and fall rates.

**Results:**

Recruitment of 10 participants was slow and took three months. Observed performance of functional abilities did not differ between groups due to the small sample size. Difference in activities of daily living participation, as recorded by the Nottingham Extended Activities of Daily Living scale, was statistically significant but wide confidence intervals and low statistical power limit interpretation of results.

**Conclusion:**

Evaluation of pre-discharge home visits by occupational therapists in a rehabilitation setting is feasible, but a more effective recruitment strategy for a main study is favored by application of a multi-centre setting.

## Background

The transition from hospital to home is often difficult for older people. A safe and successful discharge from hospital requires a person to be able to cope independently, often unsupported, in the community. One intervention that is widely believed to promote safe discharge home from acute hospital, and prevent readmission, is an occupational therapy home visit [1]. Occupational therapy home visits aim to maximise independence in the community and decrease ongoing dependency on others [2,3]. These goals are achieved by assessing a person in their own home prior to discharge, then providing equipment, adapting the home environment and/or modifying performance of daily activities. The aim of preparing someone for discharge home is to contribute to improved discharge outcomes, help maintain quality of life in older people, and potentially reduce institutionalisation and social support service involvement.

Older people value remaining at home, having autonomy and maintaining independence [4]. However, unplanned hospital re-admissions are common for older people [5,6]. This may be because older people are not always well prepared for independent living after hospital discharge, particularly the practical aspects of daily living following a change in health status. Problems can arise when an older person is discharged from hospital after an episode of inpatient care, returning home with a changed health status and increased disability. At this time, an older person may be unable to manage everyday self-care activities, and be at risk of increased falls or unplanned readmission to hospital. Anxiety about managing at home can restrict the lives of older people, and reduce community participation.

Occupational therapy intervention is recognised as an important component of successful discharge planning for older people [7]. Occupational therapists assess how older people will manage at home after discharge [8]. A visit to the older person's home prior to discharge is believed to increase the person's ability to cope at home and in the community. While studies have demonstrated that occupational therapists can identify and address potential hazards, adapt the environment and reduce falls [9-11] – the effectiveness of pre-discharge home visits remains uncertain [3,12].

One clinical trial of pre-discharge home visit efficacy has been conducted [13], however, that study was underpowered and had other methodological limitations. As a consequence there are no evidence based clinical guidelines on pre-discharge home visits. There is, however, research to indicate that occupational therapy home visits conducted *after *discharge or with community-living older persons are able to reduce the risk of falls, improve functional mobility, encourage the resumption of important life roles, increase functional independence and quality of life, and reduce the burden on carers [10,14-17].

Despite a lack of research on pre-discharge occupational therapy home visits, they are provided routinely in hospitals across Australia and overseas. Internationally, up to 50% of patients over 65 years of age receive a home visit prior to discharge from hospital [12]. These visits are often used to determine when and if a frail older person should return to their own home. One cohort study involving older people with dementia found that 84% of problems identified on a home visit were potentially serious (for example, falls risk and social isolation), but had not been identified during a hospital-based assessment [18].

Pre-discharge occupational therapy home visits are, however, expensive when compared to hospital-based assessments. City visits take an average of 108.4 minutes, including travel time [19], and visits can take a full day in regional and rural areas. In Britain, 65% of occupational therapists conduct between 11 and 40 visits per month with older patients, with 11% doing more than 60 visits per month [20]. In light of the costs associated with pre-discharge home visiting, it is essential to determine whether or not such visits improve independence and participation, at home and in the community.

The primary aim of the present study is to investigate the feasibility of a randomised controlled trial in a usual clinical setting, and the secondary aim is to explore possible effects of pre-discharge occupational therapy home visits on return to normal living after discharge from hospital in older patients. Collection of pilot data is essential in the preparation of a full-scale trial involving such a complex intervention [21,22], not only to trial the pre-discharge home visit protocol, but also to make an appropriate choice of a primary outcome measure and perform a sound power-analysis.

## Methods

Research was conducted in compliance with the Helsinki Declaration. Informed consent was obtained from each participant. The local hospital research ethics committee approved the study protocol (reference number 05/17). The trial protocol was registered with the Australian Clinical Trials Register (protocol number 1206000213549).

### Subjects

All patients admitted to a metropolitan rehabilitation unit in Sydney, Australia, referred to occupational therapy, and who fulfilled the following criteria were invited to participate:

- mild or no cognitive impairment (score 4 or fewer errors on Short Portable Mental Status Questionnaire [23])

- community dwelling prior to current hospital admission and plan to return to same dwelling on discharge (non-institutional);

- aged 65 years or older; and

- no medical contraindications that would require strict adherence to equipment recommendations

### Random allocation

Ten participants were randomly allocated to either best practice occupational therapy home visit intervention (experimental) or the standard practice in-hospital assessment and education (control) group. All baseline outcomes were assessed prior to randomisation by one of five occupational and physical therapists. The allocation schedule was computer-generated and concealed in opaque, consecutively-numbered envelopes by a person not otherwise involved in the study.

Two ward occupational therapists were trained to deliver the experimental and control interventions in a manner dictated by the trial protocol.

### Intervention

The intervention (a pre-discharge home visit), was a single home-based occupational therapy session which occurred prior to discharge. The home visit aimed to maximise functional capacity and confidence in returning home. The visit included evaluation of the home environment using the Westmead Home Safety Assessment (WeHSA) [24], assessment of the participant's resources and discharge risks using the Assessment of Living Skills and Resources (ALSAR) [25,26], assessment of functional abilities using the Functional Independence Measure (FIM™) [27], and review of prescribed equipment. Education conducted during the visit focussed on safe performance of activities in and around their home, recommendations for home modifications and equipment. The home visit was conducted at the address to which participants were to be discharged.

### Control treatment

Control participants received a single in-hospital assessment and education session. This consultation involved assessment of functional abilities using FIM™ [27], home and community accessibility using the Home and Community Evaluation (HACE) [28]. In addition, an interview addressing home duties, social and leisure activities was conducted. The therapists also provided education about safe use of equipment and information about available community services. Participants who received the control treatment did not receive a home visit from any other member of staff prior to discharge.

### Outcome assessments

**Figure 1:**
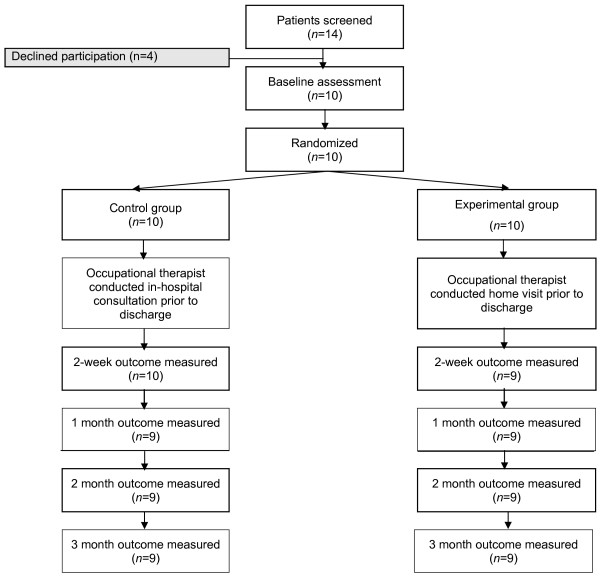
Flow of participants through the trial.

Assessments took place before the intervention, 2-weeks and one, two and three months after discharge in both groups. Figure 1 shows the course of the study and the number of participants involved at each stage. The assessor was blinded to group allocation. The outcomes measure were 1) reintegration to community living, measured using the Reintegration to Normal Living Index (RNLI) [29,30] and by asking the participant whether they got out of the house as often as they wanted (Yes/No), (2) mobility, measured by the Tinnetti Performance-Oriented Mobility Assessment [31], (3) functional status, measured by FIM™ [27] and the Nottingham Extended Activities of Daily Living scale (NEADL) [32], (4) fear of falling, measured using the Falls Efficacy Scale International scale (FES-I) [33], (5) quality of life, measured using the EQ-5D instrument [34], (6) number of falls reported by participants, (7) hours and type of community support, and (8) number of hospital readmissions.

### Analysis

Data were coded to ensure confidentiality and blinding to group allocation in the statistical analysis. To test the effects of treatment, between-group differences at each time-point were examined using ordinary least squares regression adjusting for baseline scores in analyses. P-values <.05 were regarded as statistically significant. Analysis was by intention-to-treat; missing data were omitted.

## Results

### Recruitment and sample

Recruitment took three months. Ten of 14 older people referred to the study agreed to participate. A review of admission records showed that 38 potentially eligible patients were admitted to the rehabilitation unit during the study months.

Participants were predominantly female (n = 8). Only one participant was married, one was separated, and the remaining eight were single. The majority of participants lived alone (n = 8) and did not receive services at admission (n = 7). Participants were undergoing rehabilitation for a variety of reasons, as shown in Table 1, and had an average length of inpatient rehabilitation of 25 days (range 9 to 57). There were no significant differences between the groups at baseline. Although the acute hospital length of stay prior to rehabilitation was longer in the control group, differences were not significant. All participants received the interventions as allocated, however two participants (one treatment, one control) withdrew. Reasons for withdrawal were: diagnosed with a life threatening illness unrelated to the study (n = 1); and the study assessment schedule was found to be too demanding (n = 1) (Fig. 1). Outcomes for these two participants could not be evaluated.

**Table 1:** Baseline Characteristics of Participants

	Control Group (n = 5)	Intervention Group (n = 5)
Age (years)	82.4 (7)	80.0 (7)
Gender: number of women (%)	3 (60%)	5 (100%)
Marital status: number of married (%)	1 (20%)	0 (0%)
Living situation: number living alone (%)	4 (80%)	4 (80%)
Length of Rehabilitation Stay in days (mean (SD))	25.4 (15)	25 (18)
Length of Acute Hospital Stay (prior to Rehabilitation) in days (mean (SD))	12.5 (5)	7 (3)
Home Ownership: number who owned their house (%)	4 (80%)	3 (60%)
Blaylock's Discharge Planning Risk Assessment Screen Score: score 0–40	10 (4)	9.8 (3)
Short Portable Mental Status Questionnaire number of errors (mean (SD))	1 (1)	0.8 (1)
Home Services: number receiving (%)		
Cleaning	3 (60%)	1 (20%)
Grocery Assistance	3 (60%)	0 (0%)
Gardening/Outdoor maintenance	1 (20%)	0 (0%)
Reasons for admission: number (%)		
Fall (without fracture)	3 (60%)	1 (10%)
Joint Replacement	1 (20%)	4 (80%)
Other	1 (20%)	0 (0%)

### Home Visit Intervention

The home visit intervention took a mean duration of 45 minutes (range 35 to 65) excluding travel time. The home visit plus travel time took a mean of 68 minutes (range 55 to 85). A standardised protocol ensured consistency across occupational therapists delivering the intervention. The WeHSA identified an average of four environmental hazards per visit (range 0 to 8). The ALSAR determined an average risk score for participants of 7.2 (SD 4.3; range 2 to 14) which indicates that none of the five participants who received a home visit were considered to be at risk of not accomplishing instrumental activities of daily living due to either skill or resource issues [25,26].

### Safety and Feasibility

No adverse events were recorded for participants or occupational therapists.

### Outcomes

As this was a feasibility study, the small sample dictates only the most conservative interpretation of outcomes data. Nonetheless, with the exception of the FIM™ all outcome measures showed improvements over the four outcome assessments. The RNLI, the NEADL and the EQ-5D visual analogue scale appeared most responsive to change. Further details, including mean outcomes and standard deviations, are provided in Table 2.

**Table 2:** Outcome scores and estimates of effects of all outcome measures for the control group and experimental group.

		OUTCOME SCORE		ANCOVA-ADJUSTED ESTIMATES OF EFFECTS*	p-value
Outcome Measure		Control	Experimental		

Functional Independence Measure: total score (sd), 0–126	2 weeks	115.2 (± 6.3)	117.7 (± 3.3)	2.5 (-6.7 to 11.9)	.525
	1 month	116.5 (± 7.4)	117.7 (± 3.9)	1.3 (-10.6 to 13.27)	.788
	2 months	112.2 (± 10.0)	120.0 (± 2.1)	8.72 (-3.4 to 20.9)	.125
	3 months	118.0 (± 4.0)	115.7 (± 6.8)	-0.1 (-13.9 to 13.8)	.982
Reintegration to Normal Living Index: total score (sd), 0–100	2 weeks	59.2 (± 20.3)	63.0 (± 9.6)	4.1 (-27.5 to 35.6)	.754
	1 month	56.7 (± 23.7)	56.2 (± 15.8)	0.8 (-34.8 to 36.5)	.953
	2 months	53.2 (± 38.6)	83.0 (± 9.5)	30.5 (-39.7 to 100.8)	.294
	3 months	83.6 (± 9.8)	82.0 (± 8.2)	-1.7 (-22.8 to 19.4)	.835
Nottingham Extended Activities of Daily Living scale: total score (sd), 0–66	2 weeks	37.2 (± 10.9)	45.7 (± 5.9)	16.1 (4.9 to 27.1)	.012
	1 month	33.7 (± 20.1)	46.7 (± 3.4)	17.0 (-12.88 to 46.95)	.203
	2 months	36.0 (± 13.9)	51.7 (± 3.1)	23.0 (12.2 to 33.8)	.003
	3 months	43.3 (± 7.1)	56.7 (± 2.2)	17.6 (-3.2 to 38.5)	.079
Tinnetti Performance Oriented Mobility Scale: total score (sd) 0–28	2 weeks	20.3 (± 2.9)	20.0 (± 1.4)	0.5 (-3.0 to 4.2)	.639
	1 month	22.0 (± 1.4)	24.0 (± 6.9)	2.2 (-8.9 to 13.2)	.639
	2 months	17.0 (± 11.5)	22.7 (± 2.7)	2.7 (-13.9 to 19.3)	.694
	3 months	23.0 (± 1.7)	23.5 (± 1.7)	0.5 (-3.8 to 4.8)	.779
Falls Efficacy Scale- International: total score (sd), 0–64	2 weeks	33.6 (± 7.1)	30.2 (± 8.9)	-2.3 (-18.1 to 13.5)	.735
	1 month	31.5 (± 8.0)	28.5 (± 7.3)	-2.5 (-19.8 to 14.7)	.722
	2 months	36.2 (± 9.5)	23.2 (± 4.2)	-14.8 (-30.7 to 1.17)	.063
	3 months	30.0 (± 3.0)	22.2 (± 4.5)	-3.2 (-10.5 to 4.1)	.287
EQ-5D VAS: total score (sd), 0–100	2 weeks	35.2 (± 30.8)	69.2 (± 11.3)	21.4 (-21.1 to 64.0)	.252
	1 month	62.25 (± 18.0)	79.3 (± 10.1)	8.6 (-21.4 to 38.8)	.470
	2 months	48.3(± 32.4)	87.0 (± 9.2)	31.7 (-23.4 to 86.9)	.185
	3 months	54.0 (± 25.0)	85.0 (± 8.5)	23.3 (-17.4 to 64.1)	.187

* Between-group differences adjusted for the baseline value of the outcome Larger scores reflect decreased risk except for the FES-I

#### Activities of Daily Living

The ability to perform activities of daily living as measured by the NEADL (range 0–66) was higher in the home visit group by an average of 16 points at 2 weeks, and by 23 points at 2 months (p-values .012 and .003 respectively). There were no other clinically important or significant differences between the home visit and control groups (Table 2).

#### Outings

After two weeks and also two and three months, the proportion of participants reportedly able to leave the house as often as they wanted improved in both groups from 30% (baseline proportion) to 57% or more at follow up assessments. However, there were no significant differences between the groups at any time-point.

#### Community Support Services

Overall, community support service levels decreased across both groups in the study. At discharge, seven participants (77%) were receiving community support services. This decreased to five participants at one month, and three participants at both two and three months.

#### Readmission to hospital

One participant in the control group was readmitted to hospital twice, first within two weeks of discharge and then again between the two week and one month assessments. No other participants reported being readmitted to hospital during the three month study period.

#### Falls

One of the intervention participants and one control participant reported falls within two weeks of discharge, and another control participant reported a fall at 1 month post-discharge. Two of the three falls occurred getting out of the bath, and the final fall occurred immediately after the participant had taken her medications, reporting that she felt "giddy". None of the participants sought medical attention or reported these falls to their physicians.

#### Living Situation

Living situation did not change throughout the study for any participants, with the exception of one participant in the control group who had a family member staying with her for the initial nine days post-discharge.

## Discussion

The primary aim of the study was to investigate the feasibility of conducting a randomised controlled trial involving a complex intervention, using the methods and measures previously described, in a 'usual care' rehabilitation setting. Randomisation, blinding, delivery of the intervention, data collection and analysis were feasible and conducted without problems. Use of the Westmead Home Safety Scale and development of protocols for both the home visit and control treatments ensured a standardized delivery of the care program.

Recruitment in the pilot was slow and difficult. The time taken to recruit 10 older people (3 months) was prolonged primarily due to the researchers not having direct access to potential study participants, but relying on ward occupational therapists for referrals. During the study period, 38 potentially eligible patients were admitted to the hospital, nearly three times as many as that were referred to the study. It was not possible to determine the reasons for missing older people who were potentially eligible to participate. Absence of a hospital recruiter made it difficult for all potentially suitable patients to be screened early during their admission. In addition, hospital admission rates were lower than usual and this also affected recruitment. Having multiple recruitment sites could potentially minimise the impact brought about by fluctuations in admission rates.

Having multiple recruitment sites in the future trial may address both the recruitment difficulties and ensure that the experimental (and control) interventions will be delivered by a broad range of occupational therapists. A multi-centre design will not only improve the generalizability of the findings, but also ascertain if outcomes are the result of the pre-discharge home visit intervention and not the characteristics or qualities of individual therapists performing the home visits.

The pilot study sample contained a relatively homogenous group of older people with reference to diagnosis (Table 1). Participants who received the home visit also demonstrated few living skills or resource limitations, or a need for home modifications. This reflected a less at-risk sample which, for example, included people admitted for knee replacement surgery. They were not necessarily representative of older people who would be considered for a home visit prior to discharge, suggesting a referral bias. It would therefore be appropriate to study the effect in a more heterogeneous sample to obtain a representative sample of rehabilitation in-patients. A larger sample would provide a range of people with living skills and resource risks and older people more likely to need environmental modifications.

Determining how large a sample will be required for a future trial was one of the benefits of conducting the present pilot study. Sample size must be planned carefully to ensure that the research time, patient effort and costs invested in the future trial are not wasted. Methods for the determination of sample size are described in several statistics texts, such as Altman [35], Bland, [36], and Armitage, Berry and Matthews [37]. Data from the present pilot study are invaluable in allowing a more accurate estimation of both the standard deviation of the outcome variables, and the effect size of clinical importance for the power calculation in a future trial.

Compliance with completion of the outcome measures was similar for all measures however assessors reported that participants needed more help to complete some measures. In particular, several needed clarification of RNLI questions. A possible ceiling effect was evident for the RNLI based on feasibility data, suggesting a need to assess the cultural applicability and scoring of the RNLI. On the other hand, the sensitivity of the NEADL and the ability for the EQ-5D [34] to be used as a utility measure in an economic evaluation make the NEADL and the EQ-5D ideal instruments for use in a future study. A limitation of all three of these instruments is their subjective nature; the RNLI, NEADL and EQ-5D are all self-report instruments. Use of self-report instruments makes it difficult to avoid the Hawthorne effect if the purpose of a study is revealed to participants. Unblinded participants assessing their own outcomes after being informed of the different treatment options during recruitment may bias results. Literature already suggests that the likelihood of bias increases when patients have a preference for one of the treatment options, as is likely to be the case in the present study [38]. In light of these concerns, the use of an objective, performance-based instrument would be beneficial in the future trial.

The intensive outcome measurement schedule required participants to be assessed on five occasions over three months. This schedule contributed to the substantial rate of withdrawals from the study (20%). This drop-out rate is unlikely to be acceptable in a larger study. We recommend that the number of outcome assessment time-points be reduced to decrease participant burden, and consequently minimise drop-out rates and bias. This study purposefully included a greater number of outcome measures and follow-up contacts to determine feasibility for a larger definitive randomised trial. We suggest that outcomes are assessed soon after discharge (probably two weeks) and then at a later time (probably three months).

A secondary aim was to investigate the effect on function for older people who received a home visit. Differences in outcome between groups were small, with wide confidence intervals. We assume that these small differences are related to the low statistical power of this pilot study. The study was not powered to make reliable comparisons between the treatment groups. However, an important finding was that clinically important differences were seen on the NEADL, suggesting benefits of occupational therapy pre-discharge home visits. That said, NEADL measures patient perception of functional performance rather than objectively measured performance. While the NEADL appears to be responsive to change, an alternate performance-based measure may provide confirmatory results.

## Conclusion

Based on the findings of this small pilot study we make a number of recommendations for a multicentre trial. Firstly, for more rapid recruitment, admission lists should be screened by a hospital recruiter for patients who potentially meet the inclusion criteria. This strategy is likely to result in quicker referral of patients to the study. Evaluation of cost-effectiveness should be included to determine the economic utility of a longer and more intensive intervention compared to the in-hospital interview.

This study has shown that a randomised controlled trial evaluating the effect of pre-discharge home visits is feasible. A larger multicentre trial is now required to enable recruitment of an adequate sample. Such a study is needed to inform practice, given the time and costs associated with pre-discharge home visits.

## Competing interests

The author(s) declare that they have no competing interests.

## Authors' contributions

NAL participated in the design of the study, coordinated the clinical trial, performed the statistical analysis and drafted the manuscript. LC developed the home visit intervention and participated in the design of the study. AM developed the control intervention and participated in the design of the study. CL participated in the study coordination and helped to draft the manuscript. IC conceived the study and participated in its design. SB assisted in the design of the home visit and control interventions and participated in the design of the study. All authors read and approved the final manuscript.

## Pre-publication history

The pre-publication history for this paper can be accessed here:


